# Leadership among Director of Social Work at Skilled Nursing Facilities (SNFs)

**DOI:** 10.1093/geroni/igab046.3268

**Published:** 2021-12-17

**Authors:** John Paul Abenojar

**Affiliations:** Walden University, Arlington, Virginia, United States

## Abstract

Skilled Nursing Facilities (SNFs) provide ongoing care to the seniors and chronically ill. To maximize the quality of this care, SNF staff must be trained to respond to patient care crises and communicate across departments. Although researchers have studied the leadership styles, strategies, and interactions of facility administrators and nursing directors, little was known about the leadership styles and strategies imployed by the director of social worker (DSW). The aim of this phenomenological study was to explore how DSW influenced leadership policies, prepared subordinated for crisis intervention and management, perceived that social workers influenced patient care decisions, and believed that communication amongst SNF staff about patient care could be improved. The conceptual framework was based on the multilevel leadership model construct, the situational leadership model construct, and the complex adaptive leadership model construct. Participants included a purposive sample of 10 DSW working in a large, corporate SNFs in Virginia. Data were collected via in person, semi structured interviews consisting of open-ended questions. Data were analyzed via Hycner's phenomenological approach. Findings from this investigation helped clarify roles and responsibilities of DSW, thereby improving the leadership they provide to subordinate social workers. Findings may be used to improved communication across professionals within SNFs and in their role in patient decisions.

